# Spanish-Speaking Hispanic Patients’ Information-Sharing Preferences During Hospitalization: An Exploratory Pilot Study

**DOI:** 10.2196/10782

**Published:** 2018-12-06

**Authors:** Marge Benham-Hutchins, Sharon A Brown, Erin E Donovan, Henry Guevara, Alisha H Johnson

**Affiliations:** 1 School of Nursing University of Texas at Austin Austin, TX United States; 2 Center for Health Communication Moody College of Communication and Dell Medical School University of Texas at Austin Austin, TX United States; 3 Department of Communication Studies Moody College of Communication University of Texas at Austin Austin, TX United States; 4 Dell Medical School University of Texas at Austin Austin, TX United States

**Keywords:** cancer, chronic disease, diabetes, Hispanic, self-management

## Abstract

**Background:**

Self-management of chronic conditions, such as cancer or diabetes, requires the coordination of care across multiple care settings. Current patient-centered, hospital-based care initiatives, including bedside nursing handoff and multidisciplinary rounds, often focus on provider information exchange and roles but fall short of the goals of participatory medicine, which recognize the right of patients to partner in their own care and play an active role in self-management.

**Objective:**

This study aimed to elicit Spanish-speaking Hispanic patients’ perspectives on the exchange and sharing of information during hospitalization.

**Methods:**

This exploratory pilot study incorporated a qualitative descriptive approach by using Spanish language focus groups, posthospitalization, to determine patient-identified information needs during hospitalization.

**Results:**

Participants preferred paper-based Spanish language medical information. Doctors and nurses were key information providers and communicated with participants verbally, usually with the assistance of a translator. Participants expressed a desire to be informed about medication and treatments, including side effects and why there were changes in medication during hospitalization. In addition, they expressed interest in knowing about the progress of their condition and when they could expect to go home. Emotional readiness to receive information about their condition and prognosis was identified as an individual barrier to asking questions and seeking additional information about their condition(s).

**Conclusions:**

Overall, participants shared positive experiences with providers during hospitalization and the usefulness of self-care instructions. Language was not recognized as a barrier by any of the participants. Nevertheless, future research on the influence of emotional readiness on the timing of medical information is needed.

## Introduction

Current patient-centered, hospital-based care initiatives, including bedside nursing handoff and multidisciplinary rounds, often focus on provider information exchange and roles but fall short of the goals of participatory medicine, which recognize the right of patients to partner in their own care and play an active role in self-management [1,2]. Research has shown that patients who were more involved with their care had better health outcomes, fewer hospitalizations, and lower health care costs [3]. In the United States, the Patient-Centered Outcomes Research Initiative was established in 2010 to support the patient-centered care research component of the Patient Protection and Affordable Care Act [4], commonly referred to as Obamacare. The Patient-Centered Outcomes Research Initiative vision recognizes the importance of patients having “information they can use to make decisions that reflect their desired health outcomes” [5], but little is known about patient-identified information needs.

Self-management of chronic conditions, such as cancer or diabetes, requires coordination of care across multiple care settings. For example, in the outpatient environment, patients with cancer commonly participate in shared decision making, starting with treatment decisions when first diagnosed, monitoring laboratory results during chemotherapy and radiation treatments, and monitoring the efficacy of treatment and progression or remission of cancer through imaging studies [6]. Web-based ambulatory personal health record portals commonly include test results and visit summaries. Patients participating in the “Open Notes” project have real-time access to their ambulatory medical record, including clinician notes [7]. Participating patients have reported that this access helped them understand their medications and review and recall treatment decisions [7].

Hospitalization has been described as a disempowering experience [8] that can be particularly challenging for Spanish-speaking Hispanic patients as a result of cultural differences [9] and language barriers, which have been associated with patient safety risks [10,11] and misunderstandings that lead to adverse events during hospitalization and after discharge [12].

One way to support inpatient access to health information during hospitalization [13-15] is for health care providers to share medical information during interdisciplinary rounds and nursing shift change handoff at the bedside. Interdisciplinary rounds may include registered nurses, nurse practitioners, social workers, respiratory therapy, physical therapists, and multiple physicians (consultants, attending, and residents).

Sharing medical information with patients during these provider-focused events supports the exchange of health information such as treatment options, test results, care decisions, and discharge plans. Nonetheless, research has shown that patients may be reluctant to ask questions because of structural barriers, such as the way the care is delivered or organized, perceptions of paternalistic attitudes, and the power imbalance between patients and providers [16,17]. This pilot study aims to identify what health information Spanish-speaking Hispanic patients want and need during hospitalization and explore participants’ views on inclusion in nursing shift change bedside handoff and interdisciplinary rounds held in their hospital room.

## Methods

### Study Design

This exploratory pilot study incorporated a qualitative descriptive approach. Spanish language focus groups, posthospitalization, were used to elicit patient-identified information needs during hospitalization and how hospitalization influences patient self-management of cancer across care transitions. Spanish language focus groups were selected because they have been shown to create a culturally supportive environment that encourages interaction between participants and facilitators [18].

### Sample and Setting

Participants were eligible for inclusion if they were Hispanic; were Spanish-speaking; were aged ≥18 years; were living in the community; were diagnosed with cancer or another chronic disease; and have had inpatient hospitalization after their chronic disease diagnosis. Recruitment flyers with study information and research team contact information were distributed at a local cancer center, community clinics, and through the university Hispanic employee organization. In addition, Spanish-speaking team members were available through phone or at designated times at the cancer center to answer questions and sign up participants who met the inclusion criteria.

### Study Procedures

First, the research team collaborated on the development of culturally appropriate focus group questions and probes. Next, a focus group interview guide was developed by the research team with input from 2 Spanish language focus group consultants and professional focus group moderators. The guide included an introduction that provided the purpose of the session, including the reason for the focus on Spanish-speaking Hispanic patients with chronic diseases, and introduction of the main study concepts. The interview and the demographic question questionnaire were translated into Spanish and reviewed by native Spanish speakers. The focus group facilitators used the research team-developed interview questions and guide. Textbox 1 contains the English version of the moderator guide.

Focus group procedures followed established guidelines [18] and were facilitated by experienced Spanish language focus group moderators and Spanish-speaking research team members. In addition, Spanish-speaking members of the research team obtained informed consent and distributed the demographic survey. Furthermore, a research team member supervised the session recordings.

**Focus group moderator guide. Guide was translated into Spanish and focus groups were conducted in Spanish.**

**Introduction:** Role of moderator; Independent moderator, not connected to research organization; general description of research; everyone can participate—no right or wrong answers; recording audio of session; anonymity

**Introductory question:** Tell us your first name, family life, and in which hospital did you most recently receive treatment?

**Transition question 1:** How do you keep track of your health information (mediations, appointments, treatments, etc) as you go from provider to provider and location to location?

- Probe: Personal health record, notebooks, electronic apps, calendars, other family member. What do you like about this method? Explain why some methods have not worked for you.

**Transition question 2:** What about when you are in the hospital during a hospital stay? How do you keep track of your health information (what the providers tell you about your medications, tests, and treatment plan) while you are in the hospital?

- Probe: In-patient portal (ask if they know what this is and explain what this is); white board in the room; personal tools—paper and pencil (eg, notebook; folders); electronic method, family member. What about this method works best for you? What does not work for you?

**Key question 1:** Tell me about the last time you were hospitalized. Who explained your self-management treatment to you? How did the conversation make you feel?

- Probe: Describe the conversation? Were you invited to participate in the discussion? Did they speak Spanish or did they have a translator? If you asked a question, did you feel that the personnel were responsive?

**Key question 2:** What kinds of medical information are you interested in knowing about or documenting when you are in the hospital? Explain why this information is important to you?

- Probe: Is there anything you would prefer not to know about when you are in the hospital? What information do you need while hospitalized to prepare you for managing your own care when you go home?

**Key question 3:** Could you tell me about a time when you got information in the hospital that really helped you with self-care after you went home?

- Probe: What about a time when things didn’t go so well, what happened there and what do you think contributed to its failure? Do you think because you have trouble speaking English that you are treated differently (better or worse)?

**Key question 4:** When you first got home from the hospital, how confident were you that you knew what you would need to do to take care of your medical needs? Did you stop to think about whether you had all the information you thought you’d need? Did you know what to watch out for to recognize problems and what to do about it?

- Probe: What examples come to mind, good or bad? What barriers, if any, did you encounter? Did you feel you were given enough information to properly care for yourself?

**Ending question:** What factors do you think encourage patient participation in conversations about self-care? What factors do you think discourage patient participation in self-care?

- Probe: Does culture or language play a role? If so, in what way? What, if anything, should be done for those who are Spanish speakers? If you were in charge of the hospital and you were asked to redesign the self-care process to improve it, what would you do? (Probe: newer technology, better patient/doctor interactions) What would you do specifically to help Spanish speakers?

**End Session**

### Measures

Demographic questions included participants’ age, education, gender, ethnicity, primary chronic disease, secondary chronic diseases, hospitalization date, and length of stay. During the focus group, participants were also asked to recall hospital characteristics and information-sharing processes (eg, nursing handoff location and patient invitation to participate). Focus group questions included asking about patients’ beliefs regarding existing self-management practices, the influence of hospitalization on self-management, patient information access during hospitalization, and information needed by patients to resume self-management after discharge.

### Data Analysis

Descriptive statistics were used to analyze the demographic questions. Focus group sessions were conducted in Spanish, audiotaped, transcribed, and translated into English by Spanish language focus group moderators. The research team members and focus group moderator were debriefed after each session. Analysis of the focus group transcripts followed the steps of conventional content analysis [19,20]. For this study, the focus group transcripts were translated from Spanish to English, and the analysis was completed on the English language versions.

### Protection of Human Subjects

The University of Texas at Austin Institutional Review Board approval and cancer center permission were obtained before the study commenced. Spanish language informed consent was obtained per regulatory guidelines and institution institutional review board approvals. Participation was voluntary, and participant identification information was not collected during the focus groups. A US $75 gift card was offered as an incentive for participants’ time and transportation.

## Results

### Characteristics of the Focus Group Participants

The 2 focus groups, one with 6 and another with 2 participants, were conducted at the School of Nursing on the campus of the University of Texas at Austin. Focus group participants (*n*=8) were Hispanic women with an average age of 55 (range: 47-66) years. While primary chronic disease diagnoses included cancer and diabetes, secondary chronic diseases included hypertension, arthritis, idiopathic thrombocytopenia purpura, and heart or liver disease. Participants reported having been hospitalized within the last 2-8 months and were hospitalized for 2-14 days (µ=6).

### Qualitative Results

The English translation versions of the focus group transcripts were used for analysis. We identified 5 categories as follows: tracking health information; opportunities to participate in information exchange; information needs and desires; information shared by providers during hospitalization; and self-care and self-efficacy.

### Tracking Health Information

Participants were asked about how they kept track of their health information (medications, appointments, treatments, etc) across multiple care settings and providers. Most participants did not have a specific method for keeping track, but some relied on “papers” handed to them by providers and on reminder calls initiated by the providers. Most did not have home computers or internet access but did have the ability to text using their mobile phones. When discharged from the hospital, participants reported receiving a folder or a bag with information about their condition and upcoming treatments. This information was commonly provided in both English and Spanish. When asked about reading the information, participants indicated a preference for Spanish language information, but 1 participant reported purposely not reading any of the information because she preferred not to know about her condition.

Next, participants were asked how they kept track of their health information while they were hospitalized. Results of tests, information on treatments, and medications were provided to patients, family members or relatives, almost exclusively in verbal format by nurses or other health care providers. Some participants reported receiving laboratory results and information about their treatment from their doctor in English, but always having a translator available either in-person or via telephone to assist. Furthermore, participants mentioned that they occasionally had a Spanish-speaking nurse or they relied on family members who spoke English to translate.

Participants reported that they did not actively keep track of test results, medications, or treatments in any written format or use any specific tracking system. Only 1 participant mentioned a family member taking notes. None of the participants reported having access to an inpatient portal during their hospitalization. Some participants mentioned a whiteboard in their room that provided the name of the doctor, nurse, nurse assistant, and medication administration times; others reported not having access to any of their health information while hospitalized.

### Opportunities to Participate in Information Exchange

Of specific interest were opportunities for participants to obtain health information during nursing bedside shift change handoff and medical rounds in patients’ room. During shift change handoff, most participants reported that both outgoing and incoming nurses were present in their room. Commonly, the nurse who was leaving introduced the new nurse and discussed the patient’s treatment plan for the upcoming shift. Most participants reported they were able to participate in these conversations. In some instances, only an introduction of the new nurse took place without much treatment discussion and, at times, no introduction took place before a shift change. In contrast, participants reported that the doctor(s) visited them after surgery and during rounds when they would explain the treatments the patients were receiving. All doctors made a point to introduce themselves. Participants felt doctors were respectful and as patients, they were able to understand and ask questions through in-person translators or via telephone. Furthermore, doctors shared information with families if they were present.

### Information Desires and Needs

Some participants in the focus group identified information that they would like to receive from practitioners including explanations regarding tests they are to undergo, test results, reasons for receiving certain medications or treatments, side effects of medications, and why there is a change in medications or the reason medications are not working. In addition, participants wanted to be informed about the progress of their condition and be informed regarding when they could expect to leave the hospital. Other participants wanted more information on the consequences of their condition (diabetes) when it was not controlled. Avoiding negative emotions and “sinking into depression after her cancer diagnosis” were concerns but the solutions varied. One patient indicated she wanted more information and another did not want to know more soon after the cancer diagnosis owing to feeling overwhelmed and depressed. Contact with social workers and counseling in Spanish were reported to have been helpful for some after leaving the hospital.

### Information Shared by Providers During Hospitalization

When asked to recall the last time they were hospitalized and information they received about managing their medical conditions after they went home, participants reported receiving good information from a doctor or nurse and that there was always access to a translator in-person or by telephone. Participants shared that the information was well explained, they were able to ask questions through the translator, and they knew whom to call if they had further questions or concerns once they got home. On discharge, they were provided with paper-based information in English and Spanish to take home and refer to for their self-care and follow-up treatment.

### Self-Care and Self-Efficacy

Most participants felt confident about their self-care at home once discharged from the hospital. They reported receiving the necessary information needed to take care of themselves after their discharge and what symptoms or signs to watch for to identify problems. Specific information that helped them with self-management after going home included limiting activity and other instructions to reduce bleeding and infections, learning about diet to control their diabetes, learning how to use a pillow as a coughing aid after surgery to extract sputum, and learning about a new prescription and its side effects for a chronic condition.

None of the participants felt that they encountered problems due to not being able to speak English well nor did they feel they had been treated better or worse because of it. One participant with diabetes expressed being very hesitant and uncomfortable about having to inject herself at home, but a family member was able to help using information and video instructions provided by the hospital. Health care providers who were friendly, polite, and who made patients comfortable and at ease were identified as contributing to patients’ willingness to ask and learn about self-management. Once at home, many participants noted that they received important self-management instructions (verbal and paper) from their pharmacists when picking up medications after treatments or hospitalization.

## Discussion

### Principal Findings

Participants did not hesitate to share their stories of diagnosis and treatment with the group and, in fact, seemed eager to share their experiences. For some of the participants, it was clearly the first time they had shared their story publicly. While the emotional consequences of undergoing diagnosis and hospitalization were not part of this study, the topic of depression came up in both focus groups. Participants shared their difficulties in coping emotionally with their medical condition. Research has shown that religious beliefs are an important coping mechanism for this population [21]. The focus group conversations were filled with religious references, such as “I prayed a lot” or “put it in God’s hands,” suggesting a strong faith belief among participants as a way of accepting or coping with their medical conditions.

Research has shown that psychosocial stress [22] and fear of cancer [23] and other chronic diseases, such as diabetes, impact readiness for learning and information-seeking behaviors. This study revealed that, for some participants, a major barrier to asking questions was their own reluctance to learn more about their condition, which some linked to their emotional response to dealing with their chronic disease.

Although language barriers have been associated with patient safety risks [14,15] and misunderstandings that lead to adverse events during hospitalization and after discharge [16], these Spanish-dominant patients did not believe that their inability to communicate in English acted as a barrier in understanding and implementing self-care because translators were always present or available either in-person or by phone. In the outpatient environment, participants identified that pharmacists often provided self-management information, which is consistent with previous research findings [24].

These findings stand in sharp contrast to earlier work that revealed patient frustration with the lack of access to information [25,26], provider behaviors that inhibited patient participation in bedside handoff [26], and desire for electronic access to medical records. While differences in patient demographics between the studies may explain some of the differences, there is a possibility that our findings reveal that health care initiatives focused on being culturally inclusive [27] and breaking down language barriers by providing translators and translation services [28] are improving the health care experience of Spanish-speaking Hispanic patients.

### Limitations

This study is one component of a larger program of research that focuses on patients’ access to information during hospitalization and how access to information influences the self-management of chronic medical conditions. This preliminary study revealed foundational information, but it is important to acknowledge study limitations when interpreting the results. An important lesson learned during this study was how federal initiatives and resulting news stories may influence recruitment and participation in focus groups. Overall, 24 people were scheduled to participate in the focus groups, but many unexpectedly dropped out or did not show up on the day of the focus groups. Reminder phone calls revealed hesitancy to attend owing to a fear of government “representatives” and a perceived threat of deportation. Research is needed to learn more about the perceived threat of deportation influences participation in research studies and willingness to access health care services.

It is important to recognize that the final pilot study sample was smaller than planned and included only women. In addition, participants were recruited from a limited geographic area within the city of Austin. Future research will focus on increasing the number of participants and expanding the recruitment area. This will include holding focus groups at multiple locations, on different days, and increasing efforts to recruit men.

### Conclusions

This study supports the need for research to elicit Spanish-speaking Hispanic patient perspectives on facilitators and barriers to obtaining the information they need during hospitalization and participation in traditional provider-focused, information-sharing activities such as handoffs and rounds. Overall, participants shared positive experiences with providers during hospitalization and the usefulness of Spanish language self-care instructions. Surprisingly, language was not recognized as a barrier by any of the participants. Future research on the influence of emotional readiness on the timing of medical information access and the pharmacists’ role in patient self-management in the outpatient setting is needed.
